# Small-bowel Diverticulosis:Imaging Findings and Review of Three Cases

**DOI:** 10.1155/2009/549853

**Published:** 2009-08-02

**Authors:** B. De Peuter, I. Box, R. Vanheste, S. Dymarkowski

**Affiliations:** Department of Radiology, University Hospitals Leuven, 3000 Leuven, Belgium

## Abstract

Complicated small-bowel diverticulosis is a rather uncommon cause of upper abdominal pain. It may lead to symptoms presenting with an acute onset or to chronic and nonspecific complaints. As the presentation is often similar to other pathologies (acute appendicitis, pancreatitis, or acute cholecystis) and in many cases diagnosis is made on basis of surgical findings, careful analysis of the imaging landmarks may be warranted to aid in the early stages of detection. In this report, we present clinical and morphological findings in three patients where small-bowel diverticulitis was surgically proven. The relevant literature is reviewed, and typical imaging properties are discussed.

## 1. Introduction

Small-bowel diverticulosis is an uncommon, acquired condition that is sporadically observed during conventional barium small-bowel barium follow-through studies. While frequently seen in duodenum, jejunal and ileal localisations are very rare. Only rarely symptoms occur unlike colonic diverticula, and the clinical significance of uncomplicated diverticula is minimal. Nevertheless, these small mucosal herniations may be the cause of occult upper gastrointestinal bleeding or may become impacted with food residue, become inflamed, and lead to an acute upper abdominal syndrome.

 The individual may present to the emergency room with a nonspecific inflammatory syndrome. Ultrasound and computed tomography will often be inferred in an attempt to narrow the differential diagnosis and demonstrate a focal inflammatory lesion. A specific diagnosis may not always be at hand, depending on the severity of the imaging findings diagnostic laparoscopy with segmental enterectomy may be required, while symptomatic treatment may suffice for less pronounced cases.

 The aim of this paper is to provide an overview of imaging findings in a small group of pathologically proven cases and to compare these results to those found in recent literature.

## 2. Methods

Review of our patient database revealed three patients with a final confirmed diagnosis of jejunal diverticulitis. All three patients (two females, one male, age 73–84) had presented to the emergency room for work-up of acute onset upper abdominal pain had undergone an abdominal ultrasound and a multidetector CT exam. 

 For CT, patients drank a 2.5% solution of hydrosoluble contrast agent (Telebrix Gastro, Guerbet) over the course of one hour preceding the CT scan. All exams were performed on either a 16- or 64-slice MDCT scanner using standard preset procedures during the portal venous phase after injection of 120 mL of nonionic intravenous contrast material (Xenetix 350, Guerbet) at a rate of 2–3 ml/s using a power injector (Envision CT Injector, Medrad, Pittsburgh, Pa, USA). Transverse and coronal slices of 3 mm were interpreted by the abdominal radiologist and later correlated with the surgical findings.

## 3. Imaging Findings

**Case 1**

**Figure 1:**
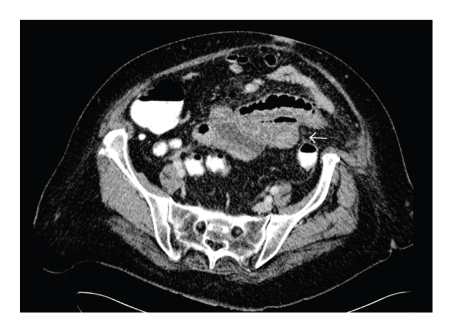
Discrete inflammatory changes in the mesogastric fatty tissue surrounding several jejunal loops. An air containing mass suggestive of a diverticulum can be seen (arrow).

A 83-year-old woman presented to the emergency room with a 3-weeks history of watery diarrhoea up to 5 times a day, dehydration, and a weight loss of 4 kg. No significant changes of the inflammatory parameters were found. The patient had a history of presacral immunocytoma treated by radiotherapy and chemotherapy. A plain radiograph and ultrasound of the abdomen at the emergency were unremarkable. The preliminary diagnosis was radio-enteritis. After 10 days of hospital the patient developed fever and an acute onset of abdominal pain. Computer tomography of the abdomen revealed 3 inflammatory mesenteric masses containing few air bubbles involving several jejunal loops and the colon transversum. Some contrast-filled and air-filled diverticula were present in the jejunum adjacent to the inflammatory mass, and the diagnosis of jejunal diverticulitis was suggested (Figure 1). At surgery a perforated jejunal diverticulum with associated abscess was found, and resection with end-to-end anastomosis was performed.

**Case 2**

**Figure 2:**
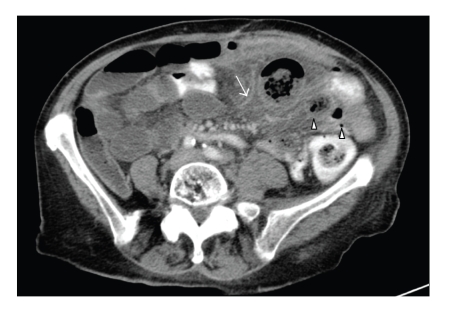
Transverse CT section through the lower abdomen reveals localised inflammatory changes in the left mesenteric root (arrow). Centrally within this pseudomass, several air containing small-bowel diverticula can be noted (arrow heads).

A 73-year-old woman presented with a 3-day history of left lower quadrant pain. Physical examination revealed left lower quadrant tenderness with rebound pain. There was a weight loss of 10 kg over 5 months. A plain radiograph was nonspecific with a few air-fluid levels. Computer tomography of the abdomen revealed multiple jejunal diverticula, surrounding inflammatory changes in the mesenteric fat adjacent to several jejunal loops with symmetric wall thickening. There were some minimal signs of pneumoperitoneum and some free intra-abdominal fluid (Figure 2). The diagnosis of jejunal diverticulitis with contained perforation was made. At surgery a resection of the involved area with primary jejunojejunal anastomosis and drainage of the abscess was performed.

**Case 3**

**Figure 3:**
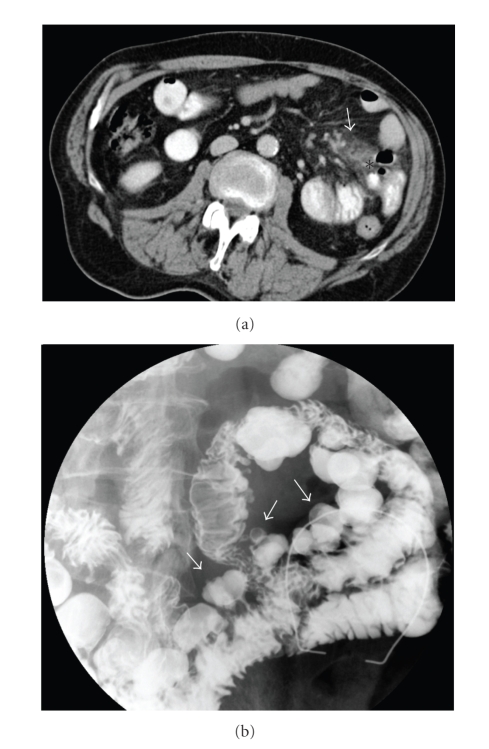
(a) Transverse CT section through the upper abdomen reveals localised inflammatory changes in the left upper quadrant (arrow) accompaigned by extraluminal air bubbles at the mesenteric side of the jejunum (asterisk). (b) Small-bowel barium follow-through exam performed three weeks after the acute onset confirms the presence of multiple jejunal diverticula (arrows).

A 74-year-old man presented to the emergency room with an acute onset of epigastric and periumbilical pain, described as being a continous pain more severe at the left side with rebound tenderness. His temperature was 39.3°C. There was no vomiting nor nausea. The patient had a history of stomach ulcer, appendectomy, and a cholecystectomy. A plain radiograph was nonspecific with a few air-fluid levels. The CT of the abdomen revealed the diagnosis of jejunal diverticulitis with limited signs of perforation (Figure 3(a)). The patient was treated with bowel rest and broad spectrum IV antibiotics. A barium study after three weeks revealed several jejunal diverticula without signs of stenosis or fistulisation (Figure 3(b)).

## 4. Discussion

Diverticulosis of the jejunum and ileum is an uncommon entity, with a reported prevalence on conventional barium studies of 0.3%–1.9% and at autopsy of 0.3%–1.3% [1, 2]. They are most common in the duodenum with a frequency of approximately 5%. They are less common in the ileum [3]. The highest incidence of jejunal diverticula is in the elderly occurring during the sixth and seventh decades of life. Jejunal diverticulitis is a very rare complication and occurs in <0.02% of cases in the general population [4]. 

 The majority of jejunal diverticula are composed of a thin wall composed of mucosal, submucosal, and serosal layer. These pseudodiverticula occur along the mesenteric border of the small bowel, usually hidden within the leaves of the mesentery. The cause of these diverticula is unclear, although it is likely that an abnormality in peristalsis, intestinal dyskinesia, and high intraluminal pressures plays a role in the pathogenesis [4]. 

 There are no pathognomonic signs or symptoms of small-bowel diverticulitis. However, they may be associated with obscure gastrointestinal bleeding or bacterial overgrowth and may on occasion become impacted with ingested food, become inflamed, and present with acute abdominal pain. Complications due to jejunal diverticula include pseudoobstruction, blind loop syndrome, jejunal dyskinesia, and chronic diverticulitis complicated by the formation of enterolith. More acute complications include perforation, peritonitis, bleeding, and fistula formation [5]. 

 At computer tomography small bowel diverticulitis usually presents as a focal area of bowel wall thickening most prominent on the mesenteric side of the bowel with adjacent inflammation and/or abcess formation. When a abcess is present, CT findings may include relatively smooth margins, areas of low attenuation within the mass, rim enhancement after IV contrast administration, gas within the mass, displacement of the surrounding structures, and edema of thickening of the surrounding fat or fascial planes.

 The differential diagnosis includes perforated neoplasm, foreign body perforation, small-bowel ulceration from nonsteroidal anti-inflammatory drug use, Crohn's disease, and diverticulitis [3]. Perforated neoplasms can be difficult to distinguish from jejunal diverticulitis. The most likely neoplasm to perforate would be lymphoma. However, lymphoma typically presents on CT as a segmental area of abnormality as opposed to a focal lesion. The findings of a gas-containing mass associated with a nearby diverticulum are suggestive of small-bowel diverticulitis. Most cases of foreign body perforations are due to an impacted fish bones or other ingested material [6]. At CT a thin linear or curvilinear density is usually noted at the site of the perforation representing the foreign body. Small bowel ulcerations from nonsteroidal anti-inflammatory drug use usually occur in the stomach or ileum but may occur anywhere in the small bowel. Although Crohn's disease usually affects the terminal ileum, isolated small bowel involvement in the jejunum may occur. Usually the process is segmental and not focal and presents with fibro-fatty proliferation, prominent vasa recta, and skip areas [7]. 

 Resection of the involved area with primary jejunojejunal anastomosis is the surgical management of choice in the presence of perforated jejunal diverticular disease, hemorrhage, or abscess formation after a failure of a short course of bowel rest and antibiotics [4].
